# A Case‐Control Study of Risk Factors for Scabies Among Patients Attending Dermatology Clinics in Afghanistan

**DOI:** 10.1002/hsr2.72918

**Published:** 2026-07-28

**Authors:** Matiullah Alimzai, Sayed Hussain Mosawi, Ahamd Jamshid Mehrpoor, Sayed Hussain Amiri, Hadia Azami, Hadia Azizi, Mohammad Saboor Siraj, Abdul Wakil Qarluq, Ioannis Adamopoulos, Aida Vafae Eslahi, Milad Badri

**Affiliations:** ^1^ Medical Sciences Research Center Ghalib University Kabul Afghanistan; ^2^ Department of Para‐Clinic, Faculty of Curative Medicine Shifa University Kabul Afghanistan; ^3^ Department of Public Health Policy, Sector of Occupational & Environmental Health, School of Public Health University of West Attica Athens Greece; ^4^ Department of Postgraduate Program in MSc Public Health Policies at Hellenic Open University School of Social Science Patra Greece; ^5^ Medical Microbiology Research Center Qazvin University of Medical Sciences Qazvin Iran; ^6^ Student Research Committee Qazvin University of Medical Sciences Qazvin Iran

**Keywords:** case–control study, neglected tropical diseases, scabies

## Abstract

**Background and Aims:**

Scabies is a highly contagious parasitic skin disease and neglected tropical disease disproportionately affecting populations in poverty and fragile health systems. In Afghanistan, evidence on its environmental and socioeconomic determinants remains scarce. This case‐control study aimed to identify demographic, socioeconomic, household, and behavioral risk factors associated with scabies among patients attending dermatology clinics in Kabul province.

**Methods:**

A clinic‐based case‐control study was conducted between March 2024 and May 2025, enrolling 410 participants (210 microscopically confirmed cases, 200 controls). Univariable and multivariable logistic regression analyses identified independent predictors of scabies.

**Results:**

In multivariable analysis, reduced bathing frequency was the strongest independent predictor (OR = 22.11, CI: 9.19–53.15 for 2–3 times/week vs. ≥ 4 times/week), followed by suboptimal personal hygiene (OR = 8.06, CI: 3.19–20.38), sharing blankets (OR = 6.43, CI: 3.20–12.92), sharing clothes (OR = 3.98, CI: 1.72–9.22), and living in a mud house (OR = 3.65, CI: 1.86–7.17 vs. apartment). Protective factors included brick housing (OR = 0.048, CI: 0.008–0.29), female gender (OR = 0.32, CI: 0.14–0.75), employment (OR = 0.31, CI: 0.11–0.84), and age ≥ 45 years (OR = 0.32, CI: 0.13–0.77).

**Conclusion:**

Scabies in Afghanistan is strongly driven by environmental deprivation, inadequate housing, poor hygiene, and material‐sharing practices. These findings underscore the need for integrated control strategies combining case management and mass drug administration with improvements in housing quality, water access, sanitation, hygiene promotion, and health education within Afghanistan's fragile health system.

## 1 Introduction

Scabies is a widespread neglected parasitic disease impacting public health globally [1]. The causative agent of scabies is the host‐specific human mite, *Sarcoptes scabiei var. hominis* [2]. Scabies has recently been classified as a neglected tropical disease (NTD) by the World Health Organization (WHO) [3]. Scabies contributed to 0.21% of global Disability‐Adjusted Life Years (DALY) in the Global Burden of Diseases (GBD) 2015 study, a metric that emphasizes the condition's considerable worldwide health impact [4]. Globally, the prevalence of scabies is estimated at 200–300 million individuals; however, these rates are known to fluctuate widely depending on the specific geographic region [5]. Scabies is prevalent in resource‐poor, crowded, and rural settings across the developing world. It is particularly problematic in contexts of social disruption, overcrowding, and poor personal hygiene. Within resource‐poor communities, specific groups face a heightened risk [6]. Young children, elderly, and immunocompromised people are particularly susceptible to both initial scabies infestation and its secondary complications [3, 7].

The disease is mainly transmitted via prolonged direct skin contact. The incubation period for a first‐time infestation is generally 3–6 weeks, whereas in cases of re‐infestation, it may be significantly shorter (1–3 days) [1]. A major concern is the common occurrence of secondary bacterial infections, which can lead to severe complications including sepsis, tissue necrosis, kidney problems, and rheumatic heart disease (RHD) [8, 9].

The clinical presentation of scabies is highly variable and depends on factors such as mite burden, patient age, immune status, and hygiene. In its classic form, patients develop an intensely itchy, symmetrical papular or papulovesicular rash 2–6 weeks after the initial infestation [10]. While the classical presentation of scabies features pronounced pruritus and cutaneous signs such as erythematous papules, pustules, and pathognomonic burrows the potential complications of this infestation are often overlooked or minimized in their clinical significance [11]. Scabies mites can persist off a human host for 1–1.5 days at room temperature (21°C) with moderate humidity (40%–80%), retaining their ability to infest [10].

The classic signs of scabies can be masked by thorough hygiene (“well‐groomed scabies”) or prior steroid use (“scabies incognito”). The relentless itching commonly disrupts sleep, leading to daytime fatigue, cognitive impairment, and lost work capacity. Social stigma, isolation, and depression are also frequent and serious complications [12].

The management of scabies faces several critical challenges. No vaccine or prophylactic exists, and therapeutic options have remained limited for 30 years to a few broad‐spectrum antiparasitic agents, notably topical permethrin and oral ivermectin. The efficacy of these drugs is often compromised by their brief half‐lives and inability to eradicate all parasite life stages, leading to common treatment failures. Successful cure typically requires at least two rounds of treatment, which is particularly challenging to implement in impoverished or itinerant communities [8]. Scabies is initially diagnosed based on symptoms and physical signs, with confirmation requiring microscopic visualization of the mite, its eggs, or feces [13]. However, there is a lack of accessible, rapid, and precise diagnostic methods to enable timely intervention and break the cycle of transmission [8].

In Afghanistan, there are very limited studies have reported the status of scabies and its related risk factors [11, 14, 15]. In this country, scabies represents a notable burden within the spectrum of dermatological conditions, accounting for an estimated 1.09% of all skin diseases reported in national health data, though this figure may understate localized outbreaks and higher prevalence in certain communities [16]. Studies conducted within the country have documented substantial point prevalence in specific high‑risk populations, as observed to be approximately 15% among boarding students in Helmand Province [15]. The situation involves extreme crowding together with people sleeping in shared bedding spaces which creates conditions that enable disease transmission to spread more effectively. The study results indicate that institutions in Afghanistan need to implement specific screening procedures together with mass drug treatment programs instead of relying solely on their current method of detecting cases.

Research has also found that knowledge and awareness about scabies among outpatients in cities like Jalalabad remains moderate to low, underscoring the need for enhanced health education and prevention programs [11].

Therefore, it is important to recognize the factors that may influence the epidemiology of scabies in Afghanistan to provide basic information for interventions toward the prevention and control of infestation. This case‐control study was conducted with the primary aim of identifying and quantifying the key demographic, socioeconomic, household, and behavioral risk factors associated with scabies among patients seeking care in dermatology clinics across several districts in Afghanistan.

## 2 Materials and Methods

### 2.1 Study Area, Design, and Participants

**Figure 1:**
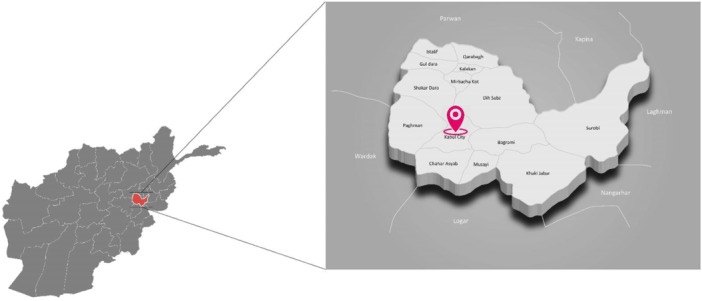
Geographic location of the study area. Map showing Kabul province within Afghanistan. All participants were recruited from dermatology clinics in this region.

This case‐control study was conducted in dermatology clinics located in Kabul Province, Afghanistan, between March 2024 and May 2025 (Figure 1). A total of 410 participants were enrolled, including 210 cases and 200 controls.

### 2.2 Data Collection and Analysis

Participants were recruited using convenience sampling. Patients meeting the eligibility criteria for either the case or control group were invited to participate, and those who provided informed consent were enrolled. Controls were frequency‐matched to cases by age group (±5 years) and clinic site. All participants underwent face‐to‐face structured interviews administered by trained interviewers at the time of clinic attendance. A formal a priori sample size calculation was not performed; therefore, the final sample size was determined by the number of eligible participants who agreed to participate during the study period.

Cases were defined as patients with a confirmed diagnosis of scabies, established by the presence of pruritic lesions consistent with scabies and microscopic visualization of mites, eggs, or scybala in skin scrapings. Exclusion criteria included prior scabicide use within 2 weeks, immunosuppression, and inability to provide informed consent.

Skin scrapings were obtained from burrows or papules using a sterile scalpel blade, placed in 10% potassium hydroxide (KOH), and examined microscopically at ×10 and ×40 magnification. A case was confirmed if at least one mite, egg, or scybala was visualized. All skin scrapings and microscopic evaluations were performed by a single dermatologist.

Controls were selected from patients attending the same clinics for non‐scabies dermatological conditions (e.g., acne, psoriasis, tinea, vitiligo, or fungal infections). They had no clinical signs of scabies and required negative microscopy from at least two skin sites (finger webs and wrists). Individuals were excluded from the control group if they had a pruritic rash, reported household contact with a scabies case within the previous 6 weeks, or had received scabies treatment within the previous 2 months.

Data were collected using a structured questionnaire covering sociodemographic characteristics, environmental and living conditions, behavioral and hygiene‐related factors, and exposure‐related variables. Sociodemographic variables included age, gender, ethnicity, education level, employment status, income, and family size. Environmental variables included residence and type of home. Behavioral and hygiene‐related variables included sleeping arrangements, blanket sharing, water source, bathing practices, personal hygiene, and history of sharing clothes. Clinical and exposure‐related variables included animal contact and travel history.

### 2.3 Statistical Analysis

The collected data were entered into Excel sheets and then exported to SPSS version 29 for analysis, where descriptive and summary statistics were performed. For the descriptive and unadjusted comparison between categorical variables, cross‐tabulation analysis using Chi‐square tests was employed. Age was also analyzed descriptively for the case and control groups. No missing data were present for any variable.

Univariable logistic regression analysis was conducted to estimate crude associations between each variable and scabies case status.

Multivariable logistic regression modeling was conducted with consideration of model stability and the possibility of overfitting. The model included 18 estimated parameters (including indicator variables for categorical predictors) for the 210 scabies cases, yielding an events‐per‐variable (EPV) of 11.7, which exceeds the often‐cited minimal requirement of 10 EPV [17]. Predictor variables were selected a priori based on clinical relevance and prior literature rather than using stepwise selection methods. Variables that displayed perfect separation (family history of scabies, contact with a scabies case, secondary bacterial infection) were excluded from multivariable estimation, as they were present almost exclusively among cases (none of the controls). In logistic regression, perfect separation prevents model convergence and leads to unstable, infinitely large odds ratio (OR) estimates.

Model fit was assessed using the Hosmer‐Lemeshow goodness‐of‐fit test. A non‐significant *p*‐value (*p* > 0.05) was considered indicative of adequate model fit.

All statistical tests were two‐sided. A *p*‐value < 0.05 was considered statistically significant. Statistical analyses were performed using SPSS version 29 (IBM Corp., Armonk, NY, USA).

## 3 Results

### 3.1 Sociodemographic Characteristics

Age distribution differed significantly between cases and controls. The mean age of the control group was 36.5 years (SD = 11.80), compared with 31.6 years (SD = 14.18) among cases, indicating that scabies cases were on average nearly 5 years younger. Participants younger than 30 years constituted a higher proportion of scabies cases (49.0% vs. 39.0% in controls), while individuals aged ≥ 45 years were less frequently affected among cases (14.3% vs. 26.0% in controls), corresponding to a crude protective effect of older age.

No significant differences were observed with respect to gender or ethnicity. Males represented approximately three‐quarters of both groups (76.8%), and Pashtun ethnicity was most common in both cases and controls (44.4%).

Education level showed a strong association with scabies. Cases were more likely to have no formal education (23.3% vs. 16.0% in controls). Notably, participants with only primary education were observed exclusively among cases (16.2%), whereas no control participant reported primary‐level education. In contrast, higher education was more prevalent among controls than cases (50.0% vs. 37.6%). Unemployment was also substantially more common among cases (32.9% vs. 14.0% in controls), representing more than a twofold crude difference. Province of residence did not show a statistically significant association (Table 1).

**Table 1:** Sociodemographic, environmental, behavioral, and clinical factors associated with scabies among cases and controls.

Variable	Categories	Control *n* (%)	Case *n* (%)	Total *n* (%)
Age group	< 30 years	78 (39.0)	103 (49.0)	181 (44.1)
	30–44 years	70 (35.0)	77 (36.7)	147 (35.9)
	≥ 45 years	52 (26.0)	30 (14.3)	82 (20.0)
Gender	Male	154 (77.0)	161 (76.7)	315 (76.8)
	Female	46 (23.0)	49 (23.3)	95 (23.2)
Ethnicity	Pashtun	88 (44.0)	94 (44.8)	182 (44.4)
	Tajik	79 (39.5)	83 (39.5)	162 (39.5)
	Hazara	33 (16.5)	33 (15.7)	66 (16.1)
Province of residence	Kabul	182 (91.0)	183 (87.1)	365 (89.0)
	Other	18 (9.0)	27 (12.9)	45 (11.0)
Education level	Higher education	100 (50.0)	79 (37.6)	179 (43.7)
	High school	68 (34.0)	48 (22.9)	116 (28.3)
	Primary school	0 (0.0)	34 (16.2)	34 (8.3)
	No formal education	32 (16.0)	49 (23.3)	81 (19.8)
Employment status	Employed	172 (86.0)	141 (67.1)	313 (76.3)
	Unemployed	28 (14.0)	69 (32.9)	97 (23.7)
Type of settlement	Apartment	103 (51.5)	90 (42.9)	193 (47.1)
	Brick	59 (29.5)	2 (1.0)	61 (14.9)
	Mud	38 (19.0)	118 (56.2)	156 (38.0)
Area of sleep	Bed	90 (45.0)	53 (25.2)	143 (34.9)
	Floor	110 (55.0)	157 (74.8)	267 (65.1)
Shared blanket	No	169 (84.5)	97 (46.2)	266 (64.9)
	Yes	31 (15.5)	113 (53.8)	144 (35.1)
Water source	Filtered	38 (19.0)	76 (36.2)	114 (27.8)
	Unfiltered	162 (81.0)	134 (63.8)	296 (72.2)
Frequency of bath	≥ 4/week	99 (49.5)	27 (12.9)	126 (30.7)
	2–3/week	37 (18.5)	116 (55.2)	153 (37.3)
	≤ 1/week	64 (32.0)	67 (31.9)	131 (32.0)
Family size	≤ 5	39 (19.5)	21 (10.0)	60 (14.6)
	> 5	161 (80.5)	189 (90.0)	350 (85.4)
Personal hygiene*	Adequate	184 (92.0)	159 (75.7)	343 (83.7)
	Suboptimal	16 (8.0)	51 (24.3)	67 (16.3)
Family history of scabies	No	200 (100.0)	78 (37.1)	278 (67.8)
	Yes	0 (0.0)	132 (62.9)	132 (32.2)
History of sharing clothes	No	182 (91.0)	151 (71.9)	333 (81.2)
	Yes	18 (9.0)	59 (28.1)	77 (18.8)
Contact with animals	No	176 (88.0)	181 (86.2)	357 (87.1)
	Yes	24 (12.0)	29 (13.8)	53 (12.9)

*Note:* *Suboptimal personal hygiene was defined based on a composite of three self‐reported practices: (a) not bathing at least every other day during the week preceding interview, (b) not washing hands with soap before meals, and (c) not washing hands after defecation. Participants reporting ≥ 2 of these were classified as suboptimal.

Table 1 presents the distribution of all studied variables comparing 210 scabies cases and 200 controls.

### 3.2 Environmental and Living Conditions

Type of settlement was strongly associated with scabies. A majority of cases resided in mud houses (56.2% vs. only 19.0% of controls), whereas brick and apartment housing were more common among controls. Similarly, sleeping on the floor was more frequent among cases (74.8% vs. 55.0% of controls). Sharing blankets was markedly higher among cases (53.8% vs. 15.5% of controls), corresponding to a crude OR of approximately 6.4 (see univariable analysis). Family size also differed: 90.0% of cases came from households with more than five members, compared with 80.5% of controls (Table 1).

### 3.3 Behavioral and Hygiene‐Related Factors

Several hygiene‐related behaviors showed strong associations with scabies. Cases were substantially less likely to bathe frequently (≥ 4 times per week: 12.9% vs. 49.5% of controls), indicating a nearly fourfold lower frequency of regular bathing in the case group. Suboptimal personal hygiene was reported by nearly one‐quarter of cases (24.3% vs. 8.0% of controls).

A history of sharing clothes was also significantly more common among cases (28.1% vs. 9.0% of controls) (Table 1).

### 3.4 Clinical and Exposure‐Related Factors

A strong association was observed between scabies and a family history of the disease. Nearly two‐thirds of cases (62.9%) reported a family history of scabies, whereas none of the controls did. Water source also differed significantly, with cases more frequently reporting the use of filtered water (36.2% vs. 19.0% of controls). Contact with animals was not significantly associated with scabies (Table 1).

Clinical cutaneous features among cases: Among the 210 scabies cases, typical scabies features were seen in 101 (48.1%), secondary bacterial infection (impetigo/pyoderma) in 45 (21.4%), inflammatory lesions (papules, pustules or vesicles) in 51 (24.3%) and excoriation‐related lesions in 6 (2.9%). No case had these findings among controls.

**Figure 2:**
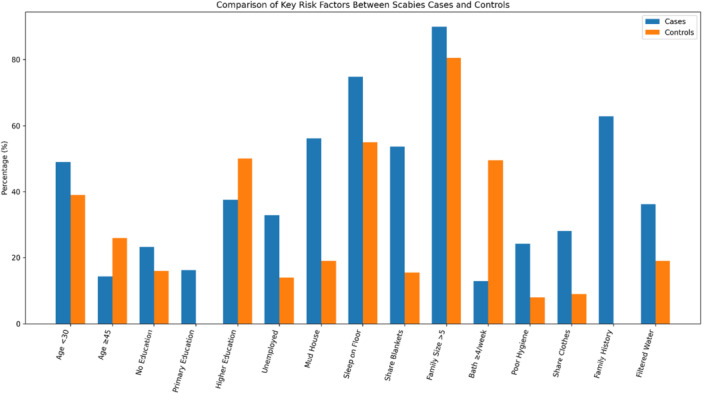
Comparison of key variables between scabies cases (*n* = 210) and controls (*n* = 200). The bar chart shows the percentage distribution of selected risk and protective factors. Cases (dark bars) consistently show higher proportions of mud housing, blanket sharing, clothes sharing, unemployment, lower income, infrequent bathing (2–3 times/week or less), and suboptimal personal hygiene compared with controls (light bars). Conversely, controls show higher proportions of brick housing, frequent bathing (≥ 4 times/week), adequate hygiene, and employment.

Cases consistently showed higher proportions of risk factors (mud housing, blanket sharing, clothes sharing, unemployment, lower income, infrequent bathing, and suboptimal hygiene) and lower proportions of protective factors (brick housing, frequent bathing, and adequate hygiene) compared with controls (Figure 2).

**Figure 3:**
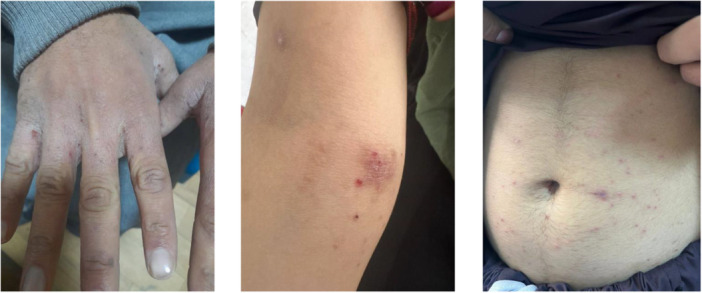
Anatomical distribution of scabies lesions among 210 cases. The figure indicates the frequency of lesions at each body site. The most commonly affected sites were the finger webs, wrists, and abdomen, which is consistent with the classic distribution of scabies in adults. Other involved sites included elbows, buttocks, and the genital area.

The most frequently affected sites were the finger webs, wrists, and abdomen, which is consistent with the classic distribution of scabies in adults. Other involved sites included elbows, buttocks, and the genital area (Figure 3).

### 3.5 Univariable and Multivariable Analysis of Scabies Risk Factors

Univariable logistic regression identified several factors significantly associated with case status. Among demographic and socioeconomic variables, lack of formal education (OR = 1.41, 95% CI: 1.18–1.68) and lower income (OR = 2.73, 95% CI: 1.74–4.28) were associated with increased odds, whereas employment was associated with lower odds of scabies (OR = 0.33, 95% CI: 0.20–0.54) showed notable associations.

Household and behavioral factors such as living in a mud house (OR = 1.75, 95% CI: 1.40–2.18), sleeping on the floor (OR = 2.42, 95% CI: 1.60–3.68), sharing blankets (OR = 6.35, 95% CI: 3.97–10.15), larger family size (OR = 2.18, 95% CI: 1.23–3.86), reduced bathing frequency (OR = 1.82, 95% CI: 1.41–2.35), suboptimal personal hygiene (OR = 3.69, 95% CI: 2.02–6.72), and sharing clothes (OR = 3.95, 95% CI: 2.23–6.99) were all significant predictors, with sharing blankets and clothes showing the largest crude effect sizes (Table 2). In contrast, gender, ethnicity, travel history, and animal contact showed no significant associations. Table 2 shows the finding of univariable logistic regression analysis.

**Table 2:** Univariable logistic regression analysis of factors associated with scabies.

Variable	Category/Comparison	OR	95% CI	*p*‐value
Age group	Per category increase	0.69	0.53–0.89	0.004
Gender	Female vs. Male	1.02	0.64–1.61	0.936
Ethnicity	Per category	0.97	0.74–1.27	0.829
Residence	Urban vs. Rural	1.49	0.79–2.80	0.214
Education level	Per category	1.41	1.18–1.68	< 0.001
Employment status	Employed vs. Unemployed	0.33	0.20–0.54	< 0.001
Income	Higher vs. Lower	2.73	1.74–4.28	< 0.001
Type of home	Per category	1.75	1.40–2.18	< 0.001
Area of sleep	Floor vs. Bed	2.42	1.60–3.68	< 0.001
Shared blanket	Yes vs. No	6.35	3.97–10.15	< 0.001
Travel history	Yes vs. No	0.96	0.65–1.42	0.833
Water source	Protective category	0.41	0.26–0.65	< 0.001
Bath frequency	Per category decrease	1.82	1.41–2.35	< 0.001
Family size	Larger vs. Smaller	2.18	1.23–3.86	0.007
Personal hygiene	Suboptimal vs. Adequate	3.69	2.02–6.72	< 0.001
Sharing clothes	Yes vs. No	3.95	2.23–6.99	< 0.001
Clothes‐sharing contact	Yes vs. No	2.06	1.47–2.90	< 0.001
Contact with animals	Yes vs. No	1.18	0.66–2.10	0.585

*Note:* Clinical features (family history, contact with confirmed cases, cutaneous findings, inflammatory lesions, secondary bacterial infection) were excluded from risk factor modelingdue to complete or near‐complete separation.

Clinical variables such as family history of scabies, contact with scabies patients, cutaneous findings, inflammatory lesions, and secondary bacterial infection were present almost exclusively among cases, leading to complete separation in the logistic model; these were therefore not interpretable as independent risk factors in the multivariable model.

Multivariable logistic regression revealed that, after adjustment for potential confounders, the strongest independent predictors of scabies were bathing 2–3 times/week (aOR = 22.11, 95% CI: 9.19–53.15) and ≤ 1 time per week (aOR = 4.62, 95% CI: 1.96–10.87), both compared with bathing ≥ 4 times/week. Other significant predictors included suboptimal personal hygiene (aOR = 8.06, 95% CI: 3.19–20.38), sharing blankets (aOR = 6.43, 95% CI: 3.20–12.92), living in a mud house (aOR = 3.65, 95% CI: 1.86–7.17 vs. apartment), and sharing clothes (aOR = 3.98, 95% CI: 1.72–9.22). Protective factors included living in a brick house (aOR = 0.048, 95% CI: 0.008–0.29), female gender (aOR = 0.32, 95% CI: 0.14–0.75), employment (aOR = 0.31, 95% CI: 0.11–0.84), and age ≥ 45 years (aOR = 0.32, 95% CI: 0.13–0.77) (Table 3).

**Table 3:** Multivariable logistic regression analysis of independent predictors of scabies.

Variable	Category (reference)	Adjusted OR (aOR)	95% CI	*p*‐value
Age	30–44 (< 30)	0.85	0.42–1.74	0.663
	≥ 45 (< 30)	0.32	0.13–0.77	0.011
Gender	Female (male)	0.32	0.14–0.75	0.008
Education	High school (higher)	0.56	0.26–1.25	0.159
	No/Primary (higher)	2.20	0.96–5.04	0.064
Employment	Employed (unemployed)	0.31	0.11–0.84	0.022
Income	< 10,000 (≥ 10,000)	1.21	0.54–2.74	0.641
Type of home	Brick (apartment)	0.05	0.008–0.29	0.001
	Mud (apartment)	3.65	1.86–7.17	< 0.001
Area of sleep	Floor (bed)	1.45	0.75–2.80	0.269
Shared blanket	Yes (No)	6.43	3.20–12.92	< 0.001
Water source	Unfiltered (filtered)	0.29	0.14–0.60	0.001
Bath frequency	2–3/week (≥ 4/week)	22.11	9.19–53.15	< 0.001
	≤ 1/week (≥ 4/week)	4.62	1.96–10.87	< 0.001
Family size	> 5 (≤ 5)	1.98	0.78–5.01	0.151
Personal hygiene	Suboptimal (adequate)	8.06	3.19–20.38	< 0.001
Sharing clothes	Yes (No)	3.98	1.72–9.22	0.001

The model demonstrated good overall performance, with a Nagelkerke R^2^ of 0.695, a non‑significant Hosmer–Lemeshow goodness‐of‐fit test (*p* = 0.500), and an overall classification accuracy of 85.4%.

Supporting Information S1: Figure S1 provides a visual summary of the main risk factors, transmission pathways, and preventive measures related to scabies. The figure illustrates key environmental and behavioral risk factors prevalent in low‐resource settings, the biological life cycle of the scabies mite highlighting direct skin‐to‐skin transmission, and recommended prevention and control strategies. These components align with the factors identified in the present study's descriptive and regression analyses.

## 4 Discussion

This case‐control study addressed the significant risk factors associated with scabies among patients attending dermatology clinics in Afghanistan. Our findings underscore that scabies transmission is not merely a biomedical issue but is deeply embedded within a complex matrix of socioeconomic deprivation, environmental constraints, and behavioral practices. The current research revealed that the scabies infestation in studied population was strongly associated with poor hygiene practices, overcrowded living conditions, and material‐sharing behaviors, while higher socioeconomic status and better living conditions appeared protective. The identified predictors collectively portray scabies as a disease closely linked to poverty and social disadvantage, consistent with its classification as a NTD [3, 5].

Conversely, protective factors such as better housing, employment, and older age highlight the critical role of structural determinants in mitigating disease risk. These results have important implications for developing targeted, context‐sensitive public health interventions in Afghanistan and similar post‐conflict, resource‐limited settings.

The strongest independent predictor identified in our multivariable analysis was reduced bathing frequency. This finding emphasizes the fundamental role of personal hygiene in disrupting the scabies life cycle. Regular bathing with soap and water can mechanically remove mites and alleviate the pruritus that leads to scratching and secondary bacterial infections [9, 10, 12]. In Afghanistan, where access to clean water and sanitation facilities is often limited, particularly in rural and peri‐urban areas, bathing frequency becomes a direct proxy for both resource access and health literacy. Our result aligns with studies from other low‐income countries, where water scarcity has been directly correlated with higher scabies prevalence [18, 19]. This suggests that scabies control programs cannot be divorced from broader Water, Sanitation, and Hygiene (WASH) initiatives. Improving community access to safe water is not only a public health goal in itself but a critical prerequisite for effective scabies management [20, 21, 22].

Improving bathing frequency in resource‐limited Afghan settings requires feasible, culturally acceptable solutions. Where household water access is limited, community bathing facilities with clean water and soap already present in some urban mosques and schools could be expanded and linked to scabies education. Additionally, promoting solar‐heated water bags (low‐cost and widely available) may reduce barriers to bathing during cold months. Future operational research should evaluate the acceptability and impact of such interventions.

Closely related to hygiene was the significant risk posed by suboptimal personal hygiene and sharing clothes. These behaviors facilitate both direct skin‐to‐skin transmission and indirect transmission via fomites. The scabies mite can survive for 24–36 h off the human host, making shared garments, towels, and particularly bedding efficient vehicles for transmission [23, 24]. The potent risk associated with sharing blankets in our study is especially telling in the Afghan context, where cold winters and limited heating often necessitate blanket sharing within families, extending close contact over many hours during sleep. This creates an ideal environment for mite transfer. Our findings corroborate research from displacement camps, where shared bedding was a paramount risk factor, leading to explosive outbreaks [6, 22, 25]. Health education campaigns must, therefore, move beyond generic advice and address these culturally and climatically ingrained practices with feasible alternatives, such as the periodic airing and sunning of bedding.

The type of dwelling emerged as a major structural determinant. Living in a mud house was a strong risk factor, while living in a brick house was highly protective. Mud houses often correlate with lower socioeconomic status, poorer ventilation, greater crowding, and surfaces that are harder to clean effectively, all of which can promote mite persistence and human‐to‐human transmission [26, 27]. Brick or cement houses, typically representing a higher economic stratum, are associated with less crowding, better hygiene infrastructure, and overall improved living conditions. This housing‐quality gradient reflects the well‐documented social determinants of NTDs, where poverty creates a perfect ecology for disease transmission [28, 29, 30]. Interventions that focus solely on drug distribution without addressing these underlying environmental factors are likely to see rapid recrudescence.

Female gender was associated with reduced odds of scabies in the adjusted model, indicating higher odds of infestation among males relative to females. This finding should be interpreted cautiously, as evidence regarding the relationship between gender and scabies is inconsistent in the literature. A recent systematic review found only a weak overall association, with males having slightly higher odds of infestation, while also noting substantial variation across studies and settings {Gupta, 2024 #1}. The stronger protective association observed in the present study may therefore reflect contextual factors or methodological limitations rather than a true biological effect. An alternative explanation is selection bias related to healthcare‐seeking behavior or clinic attendance, as males and females may differ in their likelihood of presenting to dermatology clinics. Residual confounding from unmeasured social and behavioral factors also cannot be excluded.

Employment served as another strong protective factor, likely acting as a composite marker for higher income, education, health awareness, and the ability to afford better living conditions and treatment [31].

The lower risk among older adults may be attributed to several factors: acquired partial immunity from prior exposures, less physical contact in crowded settings (e.g., compared to children playing), or more stable and less crowded living arrangements within extended families [8, 10].

Notably, our univariable analysis found education level and income to be significantly associated with scabies, reinforcing the socioeconomic axis of disease transmission. While these variables were adjusted for in the multivariable model, their strong crude associations highlight that scabies disproportionately burdens the most marginalized. This aligns with the GBD data, which shows the heaviest scabies DALYs in the world's poorest regions [4]. The lack of significant association with variables like travel history and animal contact underscores that in this endemic, community‐based setting, transmission is driven by sustained close contact within households and neighborhoods, not by transient exposure or zoonotic reservoirs [9].

The clinical profile of cases further supports the severe burden of disease. The near‐exclusive presence of secondary bacterial infection among cases is a grave concern. Scabies‐related pyoderma, often caused by *Streptococcus pyogenes* and *Staphylococcus aureus*, can lead to severe sequelae including acute post‐streptococcal glomerulonephritis and RHD [8, 9]. In a fragile health system like Afghanistan's, these complications represent a significant hidden burden, diverting scarce resources and causing long‐term morbidity [26]. RHD, though triggered by pharyngeal infection, may be maintained by community streptococcal reservoirs where pyoderma following scabies is implicated. Uncontrolled pyoderma can result in development of cellulitis, abscess and even sepsis especially in cases of malnutrition or immune compromised subjects [32]. These complications illustrate that scabies prevention should not be seen as an objective on its own, but should rather be approached as a part of general skin care and prevention of skin bacterial disease. The synergy of using scabicides, in conjunction with topical antibiotics where possible, public education on hygiene, better access to water, is expected to produce a greater public health benefit compared to isolated scabies treatment [33].

While our multivariable analysis identified strong independent predictors, we did not calculate population attributable fractions (PAF) because our clinic‐based control group may not represent the general population exposure distribution. Future community‐based studies should estimate PAF to guide resource allocation. Nonetheless, the very high adjusted ORs for modifiable factors (e.g., reduced bathing frequency: OR = 22.11) suggest that substantial case reduction could be achieved through targeted hygiene and housing interventions.

Low‐cost interventions for scabies control in Afghanistan are proposed, and fall into several categories: the integration of scabies to NTD and community health worker platforms for diagnosis and treatment; the implementation of school‐based hygiene programs which incorporate distribution of soap, education, and family contact treatment; improvement of community access to water via WASH programs to facilitate frequent bathing; and culturally sensitive messaging regarding the sharing of bedding and clothes (such as sun‐drying of bedding and separate storage of infested items for 72 h). Surveillance for scabies should be incorporated into the existing NTD sentinel surveillance programs in high burden regions to allow the early detection of outbreaks. The above interventions are all low‐cost and can be implemented using current health system resources. In view of the 21.4% rate of secondary bacterial infection, it is possible that treatment will be cost‐saving.

This study is subject to several limitations that should be considered. First, the case‐control design is inherently susceptible to recall bias; participants with scabies may be more likely to recall and report poor hygiene practices. Second, the study used convenience sampling in a clinic‐based setting, which may introduce selection bias. As recruitment was restricted to individuals attending dermatology clinics, the sample may not be fully representative of the general population, particularly individuals with limited access to healthcare or those with milder disease who do not seek care. This limits the generalizability of the findings. Third, the adjusted OR for fewer bathing sessions (especially for 2–3 bathing sessions per week compared to four times per week, aOR = 22.11) were very high. While this may reflect a genuine and strong protective effect of regular bathing against scabies transmission, such extreme effect sizes should be interpreted with caution. Residual confounding (bathing session being a proxy for lack of easy access to water, unmonitored hygiene habits, and/or socio‐economic factors) and recall bias (cases remembering and reporting hygiene practices different than controls) could explain such an effect. Nevertheless, both bathing frequency categories below the reference level (≥ 4 times/week) remained significantly associated with increased odds of scabies after adjustment, supporting an important association between reduced bathing frequency and scabies risk.

Finally, while we controlled for several confounders, residual confounding from unmeasured factors like exact household density (persons per room), or healthcare access is possible.

Despite these limitations, our study has considerable strengths. It is one of the few analytical epidemiological studies on scabies from Afghanistan, a country with a substantial disease burden yet a paucity of local evidence. The use of a pretested questionnaire, and rigorous multivariable analysis strengthens the internal validity of the identified associations. The large sample size provided adequate power to detect significant risk factors.

## 5 Conclusions

This case‐control study described risk factors for scabies in Afghanistan. It showed that poverty and behavior shaped scabies disease burden. Detection of the strongest risk factors for scabies (less frequent bathing, poor personal hygiene, shared blanket and clothes, mud house stayed in) and protective factors (female gender, employment, old age, brick house) is important in order to implement more cost‐effective, less intrusive change in the preventive measures and health education for the Afghan people, with scabies as problem in addition to other conditions. The high number of ORs for hygiene and household factors in this study indicates that in addition to vertical disease specific approach of the scabies treatment, improvement of water, sanitation and housing are mandatory due to strong socioeconomic and cultural factors. The lack of family history and secondary bacterial infections in the control group could indicate that these factors are also under avoidable circumstances for scabies patients. It is fundamental to consider scabies as non‐clinical psychobiliary and environmental disease, and to study and develop more integrated measures for scabies control program in Afghanistan. Future research should include a more thorough assessment of community‐based interventions, including whether mass drug administration is feasible and effective, and whether hygiene promotion might reduce transmission.

## Author Contributions

S.H.M., M.B., and M.A. contributed to the conceptualization of the study. S.H.M., A.J.M., and M.S.S. were responsible for methodology development, resource provision, and study supervision. I.A., H.A., M.B., and A.V.E. performed data analysis and data curation. M.A., H.A., A.W.Q., and S.H.A. conducted patient visits, clinical examinations, and data collection. S.H.M., I.A., and M.B. contributed to the project administration. S.H.M., I.A., A.V.E., and M.B., contributed to writing the original draft and to reviewing and editing the manuscript. All authors have read and approved the final version of the manuscript. S.H.M. had full access to all of the data in this study and takes complete responsibility for the integrity of the data and the accuracy of the data analysis.

## Disclosure

All authors have reviewed and approved the final version of the manuscript and agree to its submission for publication in the International Journal of Environmental Health Research. The authors confirm that this work is original and has not been previously published elsewhere.

## Ethics Statement

The study protocol was approved by the Biomedical Ethics Committee of Ghalib University, Kabul, Afghanistan (AF.GUK.REC.1403.021), as well as Qazvin University of Medical Sciences. The study was conducted in accordance with international ethical standards, and permission was obtained from the relevant clinics and hospitals. Written informed consent was obtained from literate participants, while oral informed consent was obtained from illiterate participants prior to enrollment. All data were anonymized using unique identification codes to ensure confidentiality and protect participants' privacy.

## Consent

The authors have nothing to report.

## Conflicts of Interest

The authors declare no conflicts of interest.

## Transparency Statement

Sayed Hussain Mosawi affirms that this manuscript is an honest, accurate, and transparent account of the study being reported; that no important aspects of the study have been omitted; and that any discrepancies from the study have been explained.

## Supporting information


**Figure S1:** Schematic overview of scabies risk factors, life cycle, and prevention strategies. The figure illustrates major risk factors for scabies in low‐resource settings, the life cycle of *Sarcoptes scabiei* and its transmission routes, and key prevention and control measures including personal hygiene, environmental sanitation, early diagnosis, treatment, and public health interventions. This framework was designed with the assistance of an AI diagramming tool (Figure Labs, https://chat.figurelabs.ai/chat).

## Data Availability

The data that support the findings of this study are available from the corresponding author upon reasonable request.
