# Supplementary material for: A Case‐Control Study of Risk Factors for Scabies Among Patients Attending Dermatology Clinics in Afghanistan
Source: Health Sci Rep. 2026 Jul 28;9(8):e72918. doi: 10.1002/hsr2.72918 (PMC13412542; doi:10.1002/hsr2.72918)
Supplement: Supplementary file 1 — Figure S1: Schematic overview of scabies risk factors, life cycle, and prevention strategies. The figure illustrates major risk factors for scabies in low‐resource settings, the life cycle of Sarcoptes scabiei and its transmission routes, and key prevention and control measures including personal hygiene, environmental sanitation, early diagnosis, treatment, and public health interventions. This framework was designed with the assistance of an AI diagramming tool (Figure Labs, https://chat.figurelabs.ai/chat). [file HSR2-9-e72918-s001.pdf]

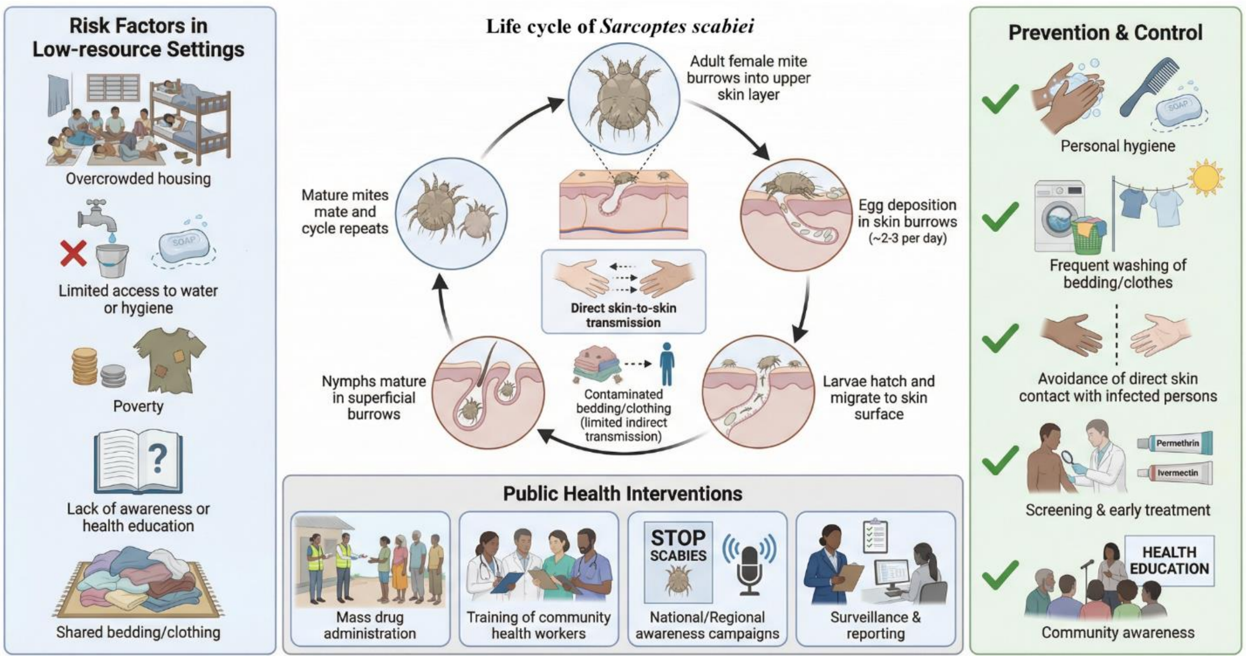

Supplementary Figure 1. Schematic overview of scabies risk factors, life cycle, and prevention strategies. The figure illustrates major risk factors for scabies in low-resource settings, the life cycle of *Sarcoptes scabiei* and its transmission routes, and key prevention and control measures including personal hygiene, environmental sanitation, early diagnosis, treatment, and public health interventions. This framework was designed with the assistance of an AI diagramming tool (Figure Labs, https://chat.figurelabs.ai/chat)
